# Flight attendant occupational nutrition and lifestyle factors associated with COVID-19 incidence

**DOI:** 10.1038/s41598-021-04350-0

**Published:** 2021-12-30

**Authors:** Jessica J. Yamamoto, Elizabeth T. Brandley, Trina C. Ulrich

**Affiliations:** Department of Health Studies, American University, 4400 Massachusetts Ave NW, Washington, DC 20016 USA

**Keywords:** Risk factors, Nutrition, Occupational health, Public health

## Abstract

In the era of COVID-19, essential workers are plagued with unforeseen and obfuscated challenges. Flight attendants are a unique subgroup of essential workers who face a multitude of health risks attributed to occupational exposures that are accentuated by the COVID-19 pandemic. Such risks can be ameliorated with strategies that target factors which enhance COVID-19 risk, including modifiable factors of diet and lifestyle. The aim of this cross-sectional study is to detect occupational dietary and lifestyle factors which could increase COVID-19 incidence amongst flight attendants. To identify potential risk factors, a questionnaire was administered to eighty-four flight attendants and examined the participants’ diet and lifestyle, and COVID-19 incidence. Descriptive statistics and logistic regression indicated that the participants’ perceived dietary quality at work (*p* = 0.003), sleep disruptions which impacted their consumption of a healthy diet (*p* = 0.013), job tenure (OR: 0.67, 95% CI: 0.46:0.98) and frequency of reported cold/flu (OR: 1.49, 95% CI: 1.014–2.189) were all factors associated with confirmed/suspected COVID-19 incidence. This study also revealed that a lack of infrastructure for food storage and time limitations are considerable occupational barriers for flight attendants to consume healthy foods. Additional investigation can further elucidate these relationships and related solutions to mitigate COVID-19 risk in the future.

## Introduction

In 2019, a novel coronavirus strain identified as SARS-CoV-2 emerged ushering in the beginning of the COVID-19 global pandemic^1^. COVID-19 is the coined name for the disease caused by the SARS-CoV-2 virus which causes a highly infectious respiratory illness that spreads rapidly from person to person via aerosols or droplets expelled by the mouth when coughing, talking, or breathing, or less commonly from contact with contaminated surfaces accompanied by subsequent facial contact^1,2^. This disease has been shown to disproportionately affect individuals with comorbid conditions such as heart disease, type 2 diabetes, hypertension, chronic obstructive pulmonary diseases, cancer, chronic kidney disease and obesity with age, race, gender, ethnicity, socioeconomic class, and residence in areas with higher air pollution being amongst other factors which have been identified to increase risks and/or severity for this infectious disease^3–14^. By mid-2021, the COVID-19 virus has impacted individuals and families across the globe with approximately 185 million confirmed cases, and approximately 4 million deaths according to the World Health Organization (WHO)^15^. These unprecedent times sent the global economy into a rapid halt in many occupational sectors due to lockdowns designed to prevent the spread of the virus^15,16^.

Essential workers were amongst those who were left on the frontlines despite global lockdowns due to their necessity in ensuring the continuing operation of critical infrastructure^17^. This unique group of workers span across a variety of professional fields including those working in health care, retail food industry, manufacturing, farming, mass transit such as buses, trains, airplanes, and beyond^17^. These professionals are often required to be in close proximity to others with frequent face-to-face interactions^18^. Additionally, they have a heightened exposure to infectious diseases and have environmental barriers which could interfere with performing healthy behaviors that mitigate illness or chronic disease^18^. Therefore, essential workers are a particularly vulnerable group^18^. Vigilant care and protection to reduce risks for infectious disease acquisition as well as the illumination of factors which may reduce risks for COVID-19 infection, including long-term complications, is needed.

Flight attendants are members of this vast group of essential workers who remain understudied yet are particularly vulnerable due to the increased rates of chronic illness compared to the general population and unique nature of their profession^19,20^. One study assessing flight attendants' health status compared to the general population from the National Health and Nutrition Examination Survey (NHANES), found both female and male flight attendants had three to five-fold greater incidence of sleep disorders, respectively^19^. This same study found that flight attendants had higher incidence of depression and fatigue symptoms^19^. Flight attendants are also at a higher risk for certain cancers^21^. In particular, a higher incidence of melanoma, non-melanoma skin cancer, and breast cancer have been found amongst female flight attendants with standard prevalence ratios being cited as 2.27, 4.09, and 1.51 respectively, compared to females from a similar socioeconomic spread^22^. The prevalence of heart disease is 3.5 times greater for female flight attendants when compared to the general public despite the fact that factors which often contribute to heart disease, such as higher biometric and metabolic markers (i.e. elevated weight and hypertension), are conditions that are reported to be lower in prevalence among flight attendants^19^. Furthermore, studies show a lower prevalence of smoking among flight attendants, yet this group is reported to have a greater incidence of chronic bronchitis and respiratory symptoms^19,20^. A recent study noted contradictory results regarding elevated chronic bronchitis risk, suggesting that this specific risk may be diminished due to removing unhealthy occupational exposures, such as mandating smoke-free environments onboard an aircraft and requires further examination^23^.

Given that COVID-19 incidence and severity rates are higher in individuals with comorbid conditions, including cancer, chronic obstructive pulmonary disease (including chronic bronchitis) and heart disease (all diseases disproportionately impacting flight attendants), it is critical to examine potential modifiable occupational factors and determinants for COVID-19 risk amongst flight attendants^24–27^.

It has been long established that healthy lifestyles that include adequate nutrition and physical activity are associated with longevity, well-being and reduced all-cause mortality; whereas lifestyles which consist of behaviors that can compromise health, such as inadequate diet, sedentary lifestyle, disordered sleep patterns, tobacco, and alcohol-use are associated with a higher incidence of a vast array of diseases and illnesses, including COVID-19^27–29^. Therefore, general recommendations for the management of COVID-19 risk have suggested adherence to healthy lifestyles^30^. Generally, adverse work life and environmental contributors to overall flight attendant health status have been cited to include noise, vibration, physical demands placed on flight attendants, and disruptions with circadian rhythm^19^. However, many of these factors may also interfere with flight attendants’ ability to consume a timely and nutrient-dense diet and adhere to healthier lifestyle patterns. The identification of areas where there is a lapse in healthy behaviors can be key to reduce one’s risks for COVID-19. Investigation into such areas of improvement in the health and wellbeing of flight attendants is lacking in current literature and warrants further attention.

To begin to address this gap in the literature, this study, to the best of the authors’ knowledge, is the first study to examine the potential occupational dietary and lifestyle determinants of COVID-19 incidence within a cohort of US based flight attendants. This study further explores the potential barriers associated with the consumption of healthy foods among flight attendants at work, compared to many of their habits at home and provides a few potential solutions. Reducing modifiable risk factors for chronic illnesses within the work environment, including targeting dietary and lifestyle measures, for the sake of the amelioration of COVID-19 risk and beyond is warranted.

## Materials and methods

### Research setting

Data for this study were collected via a research survey using Qualtrics software. The criteria for inclusion in this study included respondents must be over the age of 18, be actively employed as a flight attendant, and reside in the U.S. The survey was piloted to a small sample of flight attendants. Upon receiving feedback from this group, the updated survey was shared to social media pages specific to flight attendants, posted on public social media posts and further disseminated through targeted advertising on social media. After initial survey dissemination, to address a low respondent rate, an approved IRB amendment for an infographic to be shared with participants at the end of the survey was provided as an incentive for participation. This infographic contained specific tips for healthy eating and lifestyle suggestions for flight attendants while working and away from typical daily amenities, such as refrigeration and cooking supplies.

### Measures of variables

The survey combined a set of standardized questions (adapted and slightly modified from existing surveys), with questions specifically for flight attendants assessing their dietary needs, habits, barriers, and solutions to healthy food consumption^31,32^. The survey included questions related to demographic data and COVID-specific questions including categorical self-reported confirmed positive COVID test or suspected COVID incidence. Dietary-related questions inquiring about fruit, vegetable, and fish intake were reported in frequency ranges of daily/weekly intake. Whole grain consumption at home and at work was collected on a percentage scale from 0 to 100. The participants’ perceived dietary quality at home and at work was rated on a Likert scale from 1 to 5 (i.e., 1 = Poor, 2 = Fair, 3 = Good, 4 = Very good, and 5 = Excellent). Information on the participants’ diet type (e.g., carnivorous, vegetarian, vegan, etc.) was also collected. Additionally, information about changes in sleeping patterns when at work and the possible interference with the participants’ ability to consume a nourishing diet (i.e., “yes”, “no”, and “I don’t work in different time zones/I don’t layover in different time zones” responses) was also collected. Respondents were also asked to rate their perceived impact of the COVID-19 pandemic on their diet on a Likert scale of 1 to 5, where 1 = no effect and 5 = drastic effect. Finally, barriers associated with healthy eating at work were assessed using a list of potential responses along with a written response option.

### Data analysis procedures

Following the four-month survey administration period, data analysis was performed using SPSS V26 software. The categorical data was coded into ordinal values and was analyzed primarily using descriptive statistics with a selected level of significance of ≤ 0.05. Researchers combined suspected COVID incidence with individuals who had a self-reported or confirmed positive COVID test when analyzing the influencing variables for COVID-19 onset. Theses variables were coded as a dichotomous variable and compared with other potential predictive variables. Potential predicative variables, including the participants’ demographics such as gender, age, and job tenure, were modeled and analyzed using logistic regression. Categorical and categorical ordinal variables such as ranges of dietary intake, diet type and perceived dietary quality at home and at work were analyzed using Pearson's chi-square and Fisher’s Exact tests. The annual frequency of cold/flu incidence was also examined using logistic regression. Factors which influenced the individuals’ perceived impact that COVID-19 had on their ability to consume a healthy diet when working were also analyzed. Results were assessed with descriptive statistics (i.e., Pearson’s Chi-square and Spearman’s Correlation) to check for association between job tenure and perceived COVID-19 occupational dietary impact.

### Statement of ethical approval

This study was approved by the researchers’ university Institutional Review Board (IRB) and was performed in compliance with applicable regulations and requirements. All participants gave informed consent prior to participation.

### Ethical statement

This article is a representation of the authors’ original work and research and has not been published elsewhere.

## Results

### Demographics

A total of 84 surveys with varying completion rates were collected. As seen in Table 1, the majority of the respondents identified as cisgender female (85.7%), and the mean age of respondents fell within the 35–44 year range. The average job tenure duration was between 0 and 10 years of flying, thus indicating a higher representative sample of flight attendants with a lower job tenure in our sample population. The average monthly hours flown were 86.46 h. Approximately one-third of individuals reported having a positive COVID-19 antibody test or suspected they had the COVID-19 virus (n = 28). Additionally, 40.5% of individuals reported having 2 or more colds/flu a year (n = 34).

**Table 1:** Participant characteristics.

Characteristic	N	n (%)
Age	N = 84	
18–24 years old		7 (8.3%)
25–34 years old		26 (31.0%)
35–44 years old		18 (21.4%)
45–54 years old		19 (22.6%)
55–64 years old		12 (14.3%)
65–74 years old		2 (2.4%)
Sex	N = 84	
% Female (cisgender)		72 (85.7%)
% Male (cisgender)		10 (11.9%)
% Transgender female		0 (0%)
% Transgender male		1 (1.2%)
% Gender-fluid, non-binary		1 (1.2%)
Are you currently employed as a flight attendant?	N = 84	
Yes, I am currently actively employed as a flight attendant		72 (85.7.8%)
Yes, but I am currently on leave/furloughed		12 (14.3%)
How long have you been flying?	N = 84	
0–5 years		39 (46.4%)
5–10 years		15 (17.9%)
10–15 years		7 (8.3%)
15–20 years		5 (6.0%)
20–25 years		10 (11.9%)
25–30 years		0 (0%)
30–35 years		5 (6.0%)
35–40 years		2 (2.4%)
Over 40 years		1 (1.2%)
Have you ever tested positive for COVID-19?	N = 77	
Yes, and/or I have had a positive antibody test		11 (14.3%)
No, but I suspect that I had it		17 (22.1%)
No		49 (63.6%)
Do changes in sleeping patterns/time zones when at work interfere with your ability to consume a nourishing diet?	N = 78	
Yes		37 (47.4%)
No		35 (44.9%)
I don’t work or layover in different time zones		6 (7.7%)
During your career, how many times on average did you have a cold/flu per year?	N = 78	
0 times		11 (14.1%)
1 time		33 (42.3%)
2 times		17 (21.8%)
3 times		11 (14.1%)
4 times		2 (2.6%)
5 + times		4 (5.1%)
		**Mean (SD)**
How many hours do you fly per month		86.5 (22.6)

### Determinants which may increase COVID-19 risk

As demonstrated in Table 1, there were 77 respondents who opted to give information regarding whether they had tested positive or suspected that they had COVID-19. Slightly over half of the respondents indicated that they had not tested positive nor suspected that they had COVID-19, whereas 13% of individuals indicated that they had at least one positive COVID-19 test (or antibody test), and 20% suspected that they had COVID-19 but did not have it confirmed via testing.

A statistically significant relationship between COVID-19 confirmed/suspected incidence and job tenure was seen. While holding gender and age constant, job tenure was found to be a statistically significant predictor of COVID-19 suspected/confirmed incidence (*p* = 0.04*,* OR: 0.67, 95% CI: 0.46–0.98). Significant findings were not observed between the confirmed/suspected COVID-19 incidence and the respondents’ age (*p* = 0.562, logistic regression*)*, fruit (*p* = 0.779, Fisher’s exact*)*, vegetable (*p* = 0.15, Fisher’s exact), whole grain consumption at work (*p* = 0.883, logistic regression), and diet type (*p* = 0.669, Fisher’s Exact).

Table 2 highlights a statistically significant relationship between the respondents’ perceived diet quality at work with confirmed and suspected COVID-19 incidence (*p* = 0.003, Fisher’s exact). Those indicating a “poor” or “fair” perception of their diet at work accounted for 83% of all the positive/suspected positive COVID incidence, however, not a single participant with a suspected or confirmed COVID case rated their diet quality at work to be “very good” or “excellent”, despite respondents in these categories comprising 21% of the sample. Furthermore, although a relationship was observed between perceived dietary quality at work and COVID incidence, a statistically significant relationship was not observed between the perceived dietary quality at home and COVID incidence (*p* = 0.151, Fisher’s exact).

**Table 2:** COVID-19 incidence and perceived diet quality at work.

Qs 2. How would you rate your overall diet in terms of eating healthy foods when you are away at work/flying? (N = 52)	Qs 1. Have you ever tested positive for COVID-19?		*P* value*
	No	Yes, and/or I have had a positive antibody test/ No, but I suspect that I had it	
	N (%)	N (%)	
Poor	7 (24.1%)	5 (21.7%)	0.003
Fair	6 (20.7%)	14 (60.9%)	
Good	5 (17.2%)	4 (17.4%)	
Very good	9 (31.0%)	0 (0%)	
Excellent	2 (6.9%)	0 (0%)	
Total	29	23	

*Two-tailed Fisher–Freeman–Halton exact test.

Table 3 shows a significant relationship was found between sleep disturbances cited at work and COVID incidence. Amongst individuals who reported changing time zones when working, individuals who indicated that their sleeping patterns at work impacted their ability to consume a healthy diet appeared to have a higher suspected/confirmed COVID incidence (*p* = 0.013, X^2^ = 6.115, Cramer’s V = 0.293). Of the respondents who answered both queries and reported changing time zones at work, 72% of individuals confirmed or suspected to have experienced COVID-19 indicated that they also experienced an interference between their sleeping patterns/time zones when at work with their ability to consume a nourishing diet.

**Table 3:** Impact of COVID-19 incidence and sleep disturbance on ability to consume healthy foods.

Q2. Do changes in sleeping patterns/time zones when at work interfere with your ability to consume a nourishing diet (N = 71)	Q1. Have you ever tested positive for COVID-19?		X^2^*	*P* value
	No	Yes, and/or I have had a positive antibody test/ No, but I suspect that I had it		
	N (%)	N (%)		
No	27 (58.7%)	7 (28%)	6.115	0.013
Yes	19 (41.3%)	18 (72%)		
Total	46	25		

*Pearson’s Chi-square test.

Furthermore, as shown in Table 4, there was a statistically significant relationship found between the annual frequency of reported cold incidence and COVID incidence (*p* = 0.042, OR: 1.49, 95% CI: 1.014–2.189).

**Table 4:** Cold/flu incidence and COVID incidence.

Q2. During your career as a flight attendant, how many times on average do you have a cold/flu per year?	Q1. Have you ever tested positive for COVID-19?		OR (95% CI)	*P* value
	No	Yes, and/or I have had a positive antibody test/No, but I suspect that I had it		
	N (%)	N (%)		
0 times per year	10 (20.4%)	1 (3.6%)	1.49 (1.014–2.189)	0.042
1 time per year	21 (42.9%)	12 (42.9%)		
2 times per year	8 (16.3%)	8 (28.6%)		
3 times per year	8 (16.3%)	3 (10.7%)		
4 times per year	2 (4.1%)	0 (0%)		
5 times per year	0 (0%)	4 (14.3%)		
Total	49	28		

A perceived influence of the COVID-19 pandemic on the respondents’ ability to consume healthy foods was also observed. Participants reported that the COVID-19 pandemic impacted their dietary habits at work with a mean score of 3.3 on a 0–5 scale, with 5 indicating a drastic effect. This data suggests a skewness of − 0.544 indicating that the data wavers more heavily towards a drastic effect, than a null effect.

**Figure 1:**
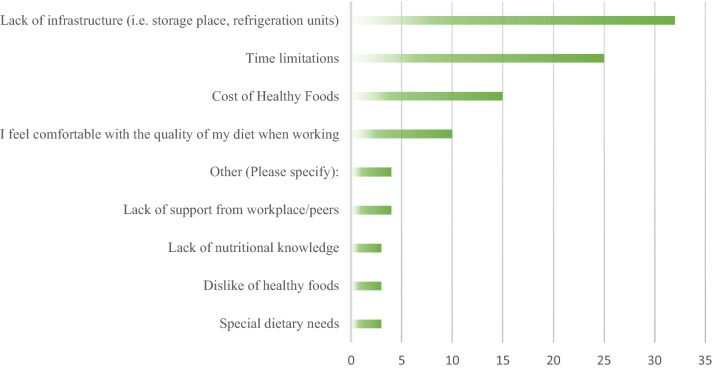
Perceived workplace barriers interfering with the introduction of healthier foods into participants’ diet.

Several barriers and solutions were also identified with regards to maintaining satisfactory dietary intake while working. When inquiring into the barriers associated with the consumption of healthy foods while working, the highest rated items were a lack of infrastructure, such as refrigeration space on the plane and on layovers, and time restrictions associated with the flight attendant work environment. Additional barriers are listed in Fig. 1.

## Discussion

This study suggests that there are modifiable dietary and lifestyle factors that can be addressed within or around flight attendants’ work environment to reduce the risk of COVID-19 and other illnesses. The significant findings between perceived influence of the COVID-19 pandemic on the quality of dietary consumption, perceived diet quality, and COVID-incidence suggests that the flight attendant workspace may be a key area for improvement among airlines to modify potential risks for COVID-19 that are rooted in sleep pattern disturbances, and/or are dietary in etiology.

It is well known that dietary intake is vital for optimal health. Eating a well-balanced diet is crucial to obtain all the daily nutrient recommendations necessary for enhanced immune health and improved overall health status^33,34^. The WHO has suggested an increased intake of fruit and vegetable consumption beyond pre-pandemic recommendations during this time period to lower the risk of potential infection^35^. However, studies assessing dietary habits during the pandemic have demonstrated that an increase in processed foods has occurred for many individuals instead^36^. Furthermore, other studies have shown that nutritional deficiencies can potentially contribute to COVID-19 infection and severity^37–39^. The findings from this study demonstrate similar results with perceived occupational dietary intake to be “poor” or “fair” being associated with positive COVID-19 incidence. Despite the workplace being a major venue for food consumption amongst working adults, there remain many questions surrounding the content of a flight attendant’s diet at work and how it supports the health of the worker^40^. One study of 5,222 adults found that nearly a quarter of all polled working adults consumed food at work, which was characteristically ripe with foods that are high in sodium and added sugars as well as solid fats^40^. These are all foods that can contribute to the onset of a variety of chronic illnesses when consumed in excess. In another study, over 30% of adults polled who purchase their meals at work, indicated that workplace cafeterias and vending machines were amongst their most frequented sites of food acquisition^41^. This suggests the potential for the worksite to be a powerful target location for the promotion of healthy behaviors which can support optimal immune function. Due to the fluid the nature of flight attendants’ work environment and limited access to healthy foods, especially during the pandemic and with their higher incidence of certain chronic diseases, it is imperative that this workgroup is supported in their efforts to consume a healthy diet to reduce risk from environmental exposures to infectious diseases and mitigate the development of chronic diseases. Since the present study is the first of its kind to illuminate occupational lifestyle factors associated with COVID-19 incidence, more research is warranted to add to the scare data on the topic to illuminate how the workplace can best be adjusted to support healthy behaviors.

Respondents reported the largest barrier preventing them from introducing more healthy foods into their diet while working was a lack of infrastructure for food storage. This highlights the need for ensuring access to reserved refrigerated storage. The solution to ensuring flight attendants have adequate storage space on board the airplane to accommodate fresh foods and meals is crucial to improving dietary intake during the pandemic and beyond. This is especially pertinent for flight attendants who have multi-day duty period and/or have extended duty period hours. Access to fresh foods at airports and even local shopping venues during layovers may be compromised due to drastic measures put in place in response to pandemic lockdown strategies, including reduced operational hours, and time for enhanced cleaning strategies^42^. Additionally, early restaurant and grocery store closures may also not be conducive to the flight schedules of late arriving flights and shorter layover durations, calling for the increased consideration on these occasions during the pandemic and in the future^43^. Additionally, early training to prepare flight attendants for certain dietary and lifestyle challenges associated with life “on the fly” in the absence of typical daily amenities is needed, however further research is warranted to investigate these health promotion efforts.

Another area of focus indicated by this study is the role of sleep disturbance connected to overall flight attendant health. This point is consistent with the current literature and presents a unique challenge to the flight attendant work group, as longer duty days and changing time zones may interfere with their ability to follow normal sleep schedules^44,45^. One study found that fatigue rooted in inadequate sleep or sleep disturbance is a common experience amongst flight attendants and affected about 84% of flight attendants polled over the course of their most recent bid period^43^. Of this group, 71% of flight attendants indicated fatigue impacting their safety-related duties. However, concerns associated with sleep and fatigue span beyond safety-related concerns. Another study which monitored the sleep habits of 202 flight attendants indicated that participants in this study had an average of 6.3 h of sleep at home, 5.7 h of sleep when working, and an even lower average when flying internationally^46^. Such sleep durations fall below the recommended 7–9 h that are suggested for optimal immune function for adults^47^. Research has demonstrated diet can impact sleep duration and quality significantly, while poor sleep can negatively influence dietary choices significantly. This is a point which was echoed by Perrin, et al. as their findings demonstrated that disruptions in circadian rhythm amongst flight attendants can subsequently interfere with established eating patterns^48–50^. Our study found similar findings between sleep disturbances interfering with diet quality and risks for positive COVID-19 incidence. Inadequate sleep has also been shown to be connected to reduced immunity function, which may also be a part of the explanation for those who had a higher incidence of confirmed or suspected COVID-19 occurrence also having a higher annual frequency of colds^51^. Solutions to these issues surrounding inadequate sleep may include a continuing focus during pandemic-times on the period of rest allotted to flight attendants during trips. This may also allow for greater opportunity to access healthy food options as after-hour food venues of convenience are often rich with energy-dense, nutrient-poor food options. Given that diet can play a role in sleep and sleep can impact dietary consumption, these two factors need to be addressed rapidly to combat the COVID-19 pandemic and beyond.

Although this study focuses on a small subset of modifiable factors with regards to COVID-19 risk amongst flight attendants, it is important to note that future studies could better inform the literature by further investigating the many factors which were not within the scope of the present paper. These established risk factors include one’s race, ethnicity and residence, factors which have been established to be risk factors for COVID-19 within the general population are of particular interest^3–14^. Further occupational factors, such as the temperate environment of airplanes, as well the air quality of selected layover destinations may also provide further information on possible risk factors for COVID-19 and subsequent measures of intervention. Temperature variation, humidity, and the presence of air pollution (especially when associated with lower wind speeds) are all factors which have been established to compromise immunity and potentially increase risks for COVID-19^13,14,52,53^. Strategic approaches to manage crisis should include preventative measures, such as the amelioration of related risk factors^54^. Therefore, the identification of further risk factors for flight attendants can help inform strategic approaches for future mitigating measures during the COVID-19 pandemic or a potential similar threat arise in the future.

## Conclusions and limitations

The novel findings of this diet and lifestyle-focused study indicate that job tenure, sleep and dietary concerns are critical factors that could contribute to COVID-19 incidence within the flight attendant population. This study also found that time limitations and a lack of reliable infrastructure to support healthy habits are significant barriers for many flight attendants. Examination into these determinants warrant further investigation for the flight attendant work group. Future studies could better support the limited literature on the topic by including a broader sample size with wider accessibility. Although access to this study survey was widely available to flight attendants in the U.S. on flight attendant-focused social media pages, which allowed for a socially distant survey administration process that was optimally accessible to flight attendants who were both actively flying, on leave, or furloughed, flight attendants who do not have access to or do not use social media are likely underrepresented in the participant pool. A larger, broader participant pool may be possible by combining mass mailing and virtual survey administration strategies. Additional studies with larger sample sizes, which explore the connection between sleep patterns and dietary intake and food access could help further illuminate the relationship between flight attendant health status and disease risk (acute and chronic).

It would be furthermore advantageous to examine dietary factors expanding beyond a dietary recall and including methods such as a food journal coupled with a food frequency questionnaire administered by trained professionals. Information on the utilization of energy-dense food venues and other food outlets and/or company provided meals and meal planning strategies would be beneficial to further the limited understanding of dietary patterns and occupational needs. A great deal can be learned from flight attendant habits, lifestyle, and disease status that can be applicable to many essential workers. Other studies can also build upon the present findings by including other work groups classified as essential workers and/or other occupational groups that have similar environmental exposures and barriers to healthy behavior choices to ensure that healthy lifestyles which support optimal immune function are feasible and attainable within one’s workspace. Investigations into possible solutions, such as increased support and adequate infrastructure such as reliable at-work refrigeration to support healthy dietary patterns is also necessary. These findings would be particularly useful as airlines navigate their way beyond the COVID-19 pandemic and incorporate preventative measures and protocols should another pandemic arise in the future.

## Data Availability

The data that support the findings of this study are available from the corresponding author upon reasonable request.
